# Hand Hygiene Knowledge, Attitudes, Practices, and Hand Dirtiness of Primary School Students Before and After a Behavioral Change Intervention During the COVID-19 Pandemic, Belize 2022–2023

**DOI:** 10.4269/ajtmh.24-0617

**Published:** 2025-05-27

**Authors:** Anh N. Ly, Christina Craig, Kelsey McDavid, Dian Maheia, Yolanda Gongora, Francis Morey, Russell Manzanero, Alexandra Medley, Allison Stewart, Allison Lino, Ramiro Quezada, Rosalva Blanco, Vickie Romero, Gerhaldine Morazan, Ella Hawes, Oluwadara Okeremi, Kanako Ishida, Matthew Lozier, Kristy O. Murray

**Affiliations:** ^1^ Department of Pediatrics, National School of Tropical Medicine, Baylor College of Medicine and Texas Children’s Hospital, Houston, Texas;; ^2^ Division of Foodborne, Waterborne, and Environmental Diseases, Centers for Disease Control and Prevention, Atlanta, Georgia;; ^3^ Belize Ministry of Education, Culture, Science, and Technology, Belmopan, Belize;; ^4^ Belize Ministry of Health and Wellness, Belmopan, Belize;; ^5^ Division of Global Health Protection, Global Health Center, Centers for Disease Control and Prevention—Central America Region, Guatemala City, Guatemala;; ^6^ Centro de Estudios en Salud, Universidad del Valle de Guatemala, Guatemala City, Guatemala;; ^7^ United States Public Health Service, Silver Springs, Maryland;; ^8^ Emory University and Children’s Healthcare of Atlanta, Atlanta, Georgia

## Abstract

Hand hygiene (HH) can prevent the spread of infectious diseases and school absenteeism. However, limited data exist on HH practices at schools. Our study assesses the impact of a pilot HH intervention in 12 schools in Belize during the coronavirus disease 2019 (COVID-19) pandemic. After a national assessment of existing water, sanitation, and hygiene resources (December 2021–January 2022), 12 pilot schools were selected to evaluate an HH intervention, which included environmental nudges and HH education. Baseline assessments occurred in March 2022, the HH intervention was implemented during October 2022–May 2023, and follow-up assessments were conducted in June 2023. Student knowledge, attitudes, practices (KAP), and hand dirtiness were assessed at baseline and follow-up. There were no changes in overall KAP median scores between the baseline and the follow-up surveys (knowledge: 3 of 4; attitudes: 11 of 12; practices: 8 of 8). There was an increase in the proportion of students who reported cleaning hands during critical moments, such as before eating and after using the restroom. Observations showed that 83% of students at baseline and 71% of students at follow-up washed their hands with soap after using the restroom. The median hand dirtiness score was seven at baseline and five at follow-up (lower score corresponds to dirtier hands). We did not observe improvements in HH after the intervention. It is possible that the decrease in perceived risk of infection as COVID-19 protocols from baseline to follow-up were reduced in schools contributed to the decrease in HH practices.

## INTRODUCTION

Hand hygiene (HH) is an effective public health intervention to prevent diarrheal and respiratory diseases in community settings, with interventions shown to reduce gastrointestinal and respiratory infections by 31% and 21%, respectively.^1^ Access to and proper use of HH resources in community settings, such as schools, is vital to protecting children’s health and ensuring an effective learning environment. Previous studies showed that HH interventions can reduce acute gastrointestinal illness-related school absenteeism by 29.5–57.1%.^2^ HH interventions in educational settings also have the potential to reduce respiratory illnesses and school absences.^3^ During the coronavirus disease 2019 (COVID-19) pandemic, the World Health Organization (WHO) recommended HH as a public health measure to prevent the spread of infections, especially during the reopening of schools.^4^^,^^5^

School-based water, sanitation, and hygiene (WASH) interventions have the potential to improve HH knowledge, attitudes, and practices among students.^6^ Though there have been mixed outcomes from school-based HH programs, depending on the intervention design, setting, and implementation, previous comprehensive school-based programs integrating infrastructure enhancement and education have yielded positive changes in HH knowledge and practices.^7^^,^^8^ To our knowledge, there has yet to be a systematic implementation and evaluation of a school-based HH intervention in Belize, an upper-middle-income country in Central America.^9^ The latest Belize-specific data from the WHO Global School-Based Health Survey in 2011 showed that 58% and 87% of students self-reported washing hands before eating and after using the restroom, respectively.^10^ However, reported HH practices may not be representative of actual practices.

Our study aimed to assess students’ HH practices through direct observation; HH knowledge, attitudes, and practices; and hand dirtiness in Belize during the COVID-19 pandemic. Our second objective was to evaluate a behavior change intervention as captured by changes in students’ observed HH practices and self-reported HH knowledge, attitudes, practices, and hand dirtiness after an intervention. The findings from this intervention can be used to inform future interventions to improve HH in schools in Belize and similar contexts, especially during a public health emergency.

## MATERIALS AND METHODS

### Study design.

A national survey was conducted electronically among all government and government-aided schools in Belize from December 8, 2021, to January 11, 2022, to collect data on existing HH infrastructure and resources at schools. Of the schools that responded (221 of 308, 72%), 12 pilot schools were selected based on the highest gaps in self-reported HH resources for further evaluation and intervention. The methods and results from the national survey, as well as an in-depth evaluation of the pilot schools’ HH resources, were published elsewhere.^11^

After the national survey, the in-depth evaluation at the 12 pilot schools included these assessments among students: survey on HH knowledge, attitude, and practices (KAP), hand dirtiness assessment, and observations of HH practices. The baseline assessments were conducted between March 8, 2022, and March 17, 2022, and the follow-up assessments were conducted between June 7, 2023, and June 15, 2023. All data were collected by enumerators who were trained in each data collection instrument, as well as human-subject research. For the hand dirtiness assessment, the enumerators practiced scoring a set of hand swabs during the training to ensure high inter-rater reliability.

### Knowledge, attitude, and practices survey.

The KAP survey was administered among students in standards I–VI (the equivalent of United States grades three to eight). If the school had multiple classrooms for each grade level, one classroom for each grade was randomly selected. Within each selected classroom, three to four students were randomly selected to participate in the survey. During the baseline assessment, classes were operating on a rotational schedule because of the pandemic (alternating grades present each day); therefore, up to eight students from each of the grades present on the day of the visit were randomly selected. Enumerators used a number generator on their mobile phones to randomly select the classrooms and students from the classrooms. At each pilot school, a maximum of 24 students were selected for the KAP survey during each assessment period. The student selections at baseline and follow-up were not paired; however, the same students may have participated in both surveys. The surveys were conducted when classes were in session throughout the school day.

A scripted overview of the study was discussed with each student, and their verbal assent to participate was obtained prior to the start of the survey. Students were informed that participation was voluntary and would not affect their grades at school. They were also informed that they could skip a question or stop participating at any time during the survey. If a student declined to participate, they returned to class, and another student was randomly selected as a replacement. Surveys were conducted on the school grounds within sight of their teacher, but at a distance from the classrooms to protect the students’ privacy when responding to questions.

Each question on the KAP survey was read aloud to the students by trained enumerators. Response options were not provided unless otherwise indicated for specific questions. Flashcards were used as visual aids for some questions to support student understanding of the response options. The responses provided by the students were recorded in Research Electronic Data Capture on tablets.^12^^,^^13^ Most surveys were conducted in English; however, the survey was administered in Spanish by native or advanced Spanish-speaking enumerators for students who did not understand English. To preserve the anonymity of the students, only grade level and sex were recorded, and each student was assigned a unique identification (ID) number. The survey took approximately 10–15 minutes to complete.

In the knowledge section of the KAP survey, students were asked questions about supplies needed to clean hands, the importance of washing hands, appropriate method of HH when hands are visibly dirty, and when they should wash their hands. In the attitude section, the questions were about students’ attitudes toward handwashing, their perception of how others view and practice HH, their preference for HH materials, and their perceived ease of access to HH materials. In the practices section, students reported their HH practices at school and at home.

### Hand dirtiness assessment.

**Figure 1.**
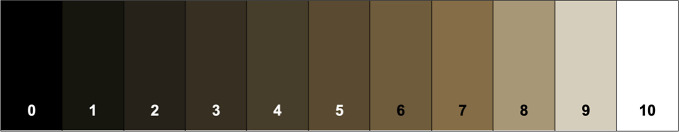
Eleven-point Quantitative Personal Hygiene Assessment Tool (qPHAT) scale.^14^

At each school, the first three students in each grade who completed the KAP survey were asked if they wanted to participate in the hand dirtiness assessment. If a student declined to participate, they returned to class, and the next student completing the KAP survey was invited to participate. A maximum of 18 hand dirtiness assessments were conducted for each pilot school at baseline and follow-up. Hand dirtiness was assessed using the Quantitative Personal Hygiene Assessment Tool.^14^ The right hand was used for all participating students. A sterile saline gauze was traced along the periphery and diagonal section of the right palm and each finger pad of the right hand. The most dense half-inch square of the swab was compared with an 11-point scale (Figure 1). The swabs were scored immediately at the school by trained enumerators. If the most dense half-inch square was between two colors on the scale, the lower score was recorded. Each hand swab was labeled with the same unique ID the student was assigned for the KAP survey to link the results of the KAP survey to the hand dirtiness results.

### Hand hygiene observations.

HH practices of students upon arriving at school and after using the restroom were observed and recorded. Observations upon arrival were either conducted at the school entrance or at the classroom entrance, depending on the location of resources at each school. For each set of observations, enumerators were instructed to record the presence of a functional HH station (handwashing station with only water, handwashing station with soap and water, and hand sanitizer dispenser), and the presence of HH informational, educational, and communication materials (posters, banners, signs, environmental nudges, etc.) at HH stations. Observations were only conducted if there was at least one of the specified HH stations available for use upon arrival or after using the restroom.

For each student observed, the following information was recorded: sex (male or female by observation), estimated age (<10 years old or ≥10 years old by observation of student height), type of HH performed (handwashing with water only, handwashing with water and soap, using hand sanitizer, or no HH performed), and presence of HH attendant. An HH attendant was defined as an adult or older student present at the HH station who reminded or assisted students with HH practices. If a student washed their hands, the duration of handwashing (<20 seconds or ≥20 seconds) was recorded. At the entrances, enumerators were instructed to observe practices of 20 students or observe for 30 minutes, whichever came first. At the restrooms, enumerators conducted 20 observations or up to 40 minutes, whichever came first. The restroom observations were conducted during school breaks and while classes were in session. To avoid influencing HH behaviors, the enumerators recorded all the observations at a distance from the HH station to be less visible to students.

### Behavior change interventions.

**Figure 2.**
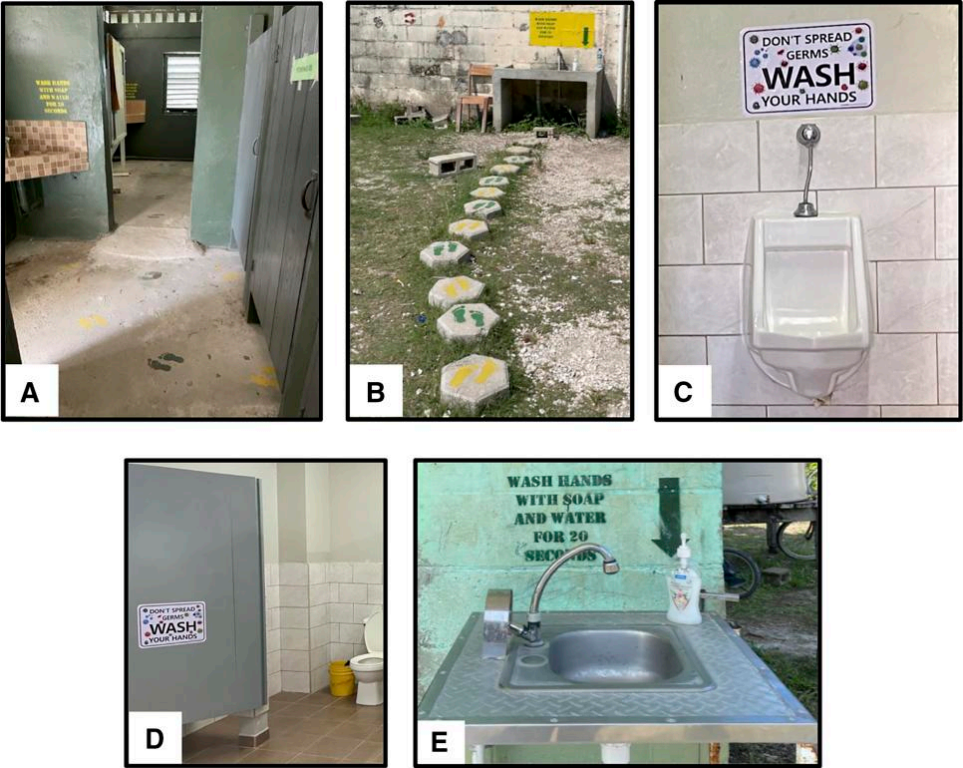
Environmental nudges for hand hygiene (HH) at schools included footsteps from each toilet stall to handwashing stations on concrete (**A**) and pavers (**B**), stickers above each urinal (**C**) or behind toilet stall (**D**), and handwashing message and arrow pointing to soap at handwashing station (**E**).

After the baseline assessment, environmental nudges were installed at schools to remind students to practice proper HH, especially after restroom use. The nudges were installed between October 2022 and February 2023. For schools with concrete flooring between toilet stalls and handwashing stations, footsteps were painted leading from each toilet stall to the nearest handwashing station (Figure 2A). At schools with grass or a dirt path between the restroom and the nearest handwashing station, concrete pavers with painted footprints were placed along the path (Figure 2B). Some schools had tile flooring inside the restrooms; therefore, the footsteps could not be painted. At these schools, a sticker with the message “Don’t spread germs, wash your hands” was placed behind each stall and above each urinal (Figure 2C and 2D). Of the 12 schools, 6 had painted footsteps, 5 had the stickers, and 1 had both painted footsteps and the stickers because they have different types of restrooms on the school compound. All HH messages were in English.

At each handwashing station where students usually wash their hands after using the restroom, a message was painted (or posted on laminated paper) on the wall: “Wash hands with soap and water for 20 seconds” (Figure 2E), and an arrow was posted on the wall beside it. Schools were instructed to place their soap bottle with the arrow pointed to it, as a reminder for students to use soap when washing hands (Figure 2E). Supplemental handwashing soap was provided to schools based on the student population in October 2022, March 2023, and May 2023.

Baylor College of Medicine (BCM) staff based in Belize and community health workers from the Ministry of Health and Wellness conducted visits to the schools in March 2023 and May 2023 to monitor the condition of the nudges. All staff were trained to use a standardized form to document their visits. Nudges were repainted if they appeared faded and schools were reminded to place soap at the handwashing stations if it was not present.

In April 2023, we conducted a workshop for school principals to develop interactive HH lessons and integrate HH concepts into different subject areas. At the workshop, principals collaboratively developed HH lesson plans for standard I through standard VI grades. Principals used the learning outcomes for each grade level as outlined by the Ministry of Education, Culture, Science, and Technology (MoECST) to determine how to best integrate HH concepts into different subject areas. The lessons were shared with the larger group for feedback during the workshop. The lessons were compiled and distributed to all school principals at the end of the workshop. Principals were instructed to deliver the lessons to teachers at their schools, and the teachers were to adapt and implement the lessons for their classes. The schools shared videos and photos of the lesson implementation and student work with study personnel at BCM. The schools were encouraged to submit videos and photos as part of a contest to award the best student work from the lessons. Of the 12 pilot schools, 9 were represented at the workshop. The lesson plans developed at the workshop were shared electronically with schools that could not be present. Documentation of lesson implementation was received from nine schools, but not all grade levels were included.

## STATISTICAL ANALYSES

The correct responses for each of the KAP questions were determined based on HH guidance from the U.S. Centers for Disease Control and Prevention and COVID-19 protocols from the MoECST. For each question, one point was awarded if the student responded with the correct answer. The sum of scores in each of the KAP sections was added to calculate three composite scores: knowledge score (4 possible points), attitude score (12 possible points), and practice score (8 possible points). Descriptive statistics were calculated to compare the distribution of student characteristics, composite KAP scores, responses to selected KAP questions, student HH practices, and hand dirtiness at baseline and follow-up. χ^2^ or Fisher’s exact test was used to assess the significance of differences in student characteristics and distribution of responses to individual KAP questions.

Bivariate multilevel logistic regression was used to assess the association between various exposure variables and three outcomes: 1) scoring at least the median score in each KAP section, 2) handwashing with soap and water after using the restroom, and 3) scoring at least the median hand dirtiness score. The HH statistical analyses were restricted to the restroom observations because the focus of the intervention was improving practices after restroom use. Multilevel logistic regression was used to account for the potential clustering effects within schools. The multivariable regression models for HH practices and hand dirtiness were constructed using the backward elimination method. All variables with a *P*-value of <0.25 in the bivariate analyses were entered into an initial model. Variables were removed one by one, starting with the variable with the highest *P*-value above 0.05. The final model contained all variables with *P*-values <0.05. In the HH practices model, HH attendant and study time point were left in the final model regardless of significance because the presence of an attendant was observed to be an important factor for student practices. All statistical tests were considered significant at the 0.05 level. Statistical analyses were performed using Stata v. 17 (StataCorp, College Station, TX).^15^

## RESULTS

Data were collected from 11 out of 12 schools at both baseline and follow-up. The one school missed at baseline (because of unplanned school staff training) was different from the one missed at follow-up (because of unforeseen long-term closure of school). A total of 209 and 234 students participated in the KAP survey at baseline and follow-up, respectively (Table 1). There were no statistical differences between the demographic characteristics of KAP participants at baseline and follow-up. There was a higher proportion of students in rural schools compared with urban schools at both baseline and follow-up.

**Table 1:** Characteristics of students participating in the KAP survey at baseline and follow-up

Student Characteristics	Baseline *N* = 209 *n *(%)	Follow-Up *N* = 234 *n* (%)	*P*-Value*
Grade			
Standards I–III	94 (45)	115 (49)	0.380
Standards IV–VI	115 (55)	119 (51)	
Sex			
Male	93 (45)	120 (51)	0.154
Female	116 (56)	114 (49)	
Locality of school			
Rural	177 (85)	189 (81)	0.277
Urban	32 (15)	45 (19)	
Region of school			
North	68 (33)	90 (38)	0.228
Central	74 (35)	66 (28)	
South	67 (32)	78 (33)	

Some column percentages do not add up to 100% because of rounding.

* *P*-values were calculated using the χ^2^ test.

The median knowledge, attitude, and practices scores did not change between baseline and follow-up: 3 of 4 for knowledge, 11 out of 12 for attitudes, and 8 of 8 possible points for practices (Table 2). There was no statistical difference in scoring at least the median knowledge and attitude scores between baseline and follow-up. Students at follow-up had lower odds of scoring at least the median score in the practices section compared with students at baseline (odds ratio [OR] = 0.65, 95% CI = 0.44–0.97). In the knowledge section, students in standards IV–VI (the equivalent of United States grades six to eight) had significantly higher odds of scoring at least the median score compared with students in standards I–III (the equivalent of United States grades three to five) (OR = 3.52, 95% CI = 1.75–7.08).

**Table 2:** The association between scoring at least the median score and student characteristics

	Knowledge*			Attitudes			Practices		
	Scored ≥ Median^†^ *n* (%)	OR (95% CI)	*P*-Value^‡^	Scored ≥ Median* *n* (%)	OR (95% CI)	*P*-Value^‡^	Scored ≥ Median* *n* (%)	OR (95% CI)	*P*-Value^‡^
Grade									
Standards I–III	174 (84)	Ref	Ref	143 (68)	Ref	Ref	116 (56)	Ref	Ref
Standard IV–VI	218 (94)	3.52 (1.75–7.08)	**<0.001**	168 (72)	1.12 (0.74, 1.70)	0.596	148 (63)	1.43 (0.97, 2.10)	0.074
Sex									
Male	189 (89)	Ref	Ref	152 (71)	Ref	Ref	120 (56)	Ref	Ref
Female	203 (91)	1.18 (0.63–2.22)	0.604	159 (69)	0.92 (0.61–1.40)	0.709	144 (63)	1.31 (0.89–1.93)	0.171
Locality									
Urban	68 (88)	Ref	Ref	47 (61)	Ref	Ref	46 (60)	Ref	Ref
Rural	324 (90)	1.23 (0.37–4.08)	0.730	264 (72)	1.68 (0.78–3.63)	0.185	218 (60)	0.98 (0.49–1.97)	0.952
Region									
North	135 (89)	Ref	Ref	109 (69)	Ref	Ref	90 (57)	Ref	Ref
Central	129 (92)	1.55 (0.50–4.74)	0.446	87 (62)	0.73 (0.41–1.32)	0.300	91 (65)	1.44 (0.78–2.65)	0.244
South	128 (88)	0.99 (0.34–2.84)	0.980	115 (79)	1.67 (0.89–3.13)	0.111	83 (57)	1.01 (0.56–1.84)	0.967
Assessment time point									
Baseline	180 (89)	Ref	Ref	155 (74)	Ref	Ref	135 (65)	Ref	Ref
Follow-up	212 (91)	1.18 (0.62–2.24)	0.612	156 (67)	0.69 (0.45–1.07)	0.095	129 (55)	0.65 (0.44–0.97)	**0.037**

OR = odds ratio; Ref = referent group.

Bold value denotes statistical significance.

* Knowledge score was calculated for 437 students.

^†^ Percentage of students who scored at least the median score within each characteristic.

^‡^ *P*-values were calculated using multilevel logistic regressions adjusting for potential clustering at the school level.

When asked about the appropriate method for HH when hands are visibly dirty, 52% of students at baseline and 47% of students at follow-up correctly answered washing with soap and water (*P*-value = 0.065) (Table 3). From baseline to follow-up, there was an increase in the proportion of students who responded that it is important to wash hands to prevent getting sick (31–43%, *P*-value = 0.009) and to get rid of bacteria, germs, and viruses (57–74%, *P*-value <0.001). A higher proportion of students reported COVID-19 as a reason to wash hands at baseline compared with follow-up (18–3%, *P*-value <0.001). The most common source of HH information for students was parents or family (85% at baseline and follow-up, *P*-value = 0.818), followed by teachers (79% at baseline and 84% at follow-up, *P*-value = 0.193). There was a 23% increase in the proportion of students who expressed that they receive HH information from posters/printed sources at schools between baseline and follow-up (*P*-value <0.001).

**Table 3:** Student responses to selected knowledge, attitudes, and practices questions* at baseline and follow-up

KAP Questions	Baseline *N* = 209 *n* (%)	Follow-Up *N* = 234 *n* (%)	Percent Change	*P*-Value^†^
Knowledge				
When your hands are visibly dirty, should you clean them with soap and water, hand sanitizer, or are both equally effective?^‡^				
Soap and water^§^	106 (52)	110 (47)	−5%	0.065
Hand sanitizer	21 (10)	13 (6)	−4%	
Both	75 (37)	107 (46)	+9%	
I don’t know	1 (1)	4 (2)	+1%	
Why is it important to wash our hands? (multiple responses)				
Prevent getting sick	65 (31)	101 (43)	+12%	**0.009**
Get rid of bacteria, germs, viruses	119 (57)	174 (74)	+17%	**<0.001**
Remove visible dirt	23 (11)	31 (13)	+2%	0.471
To not get COVID-19	38 (18)	6 (3)	−15%	**<0.001**
Where do you see or hear information about cleaning your hands? Or who has talked to you about cleaning your hands? (multiple responses)				
Parents/family	177 (85)	200 (85)	No change	0.818
Teachers	165 (79)	196 (84)	+5%	0.193
Classmates/friends	70 (33)	98 (42)	+9%	0.069
Posters/printed information at school	75 (36)	139 (59)	+23%	**<0.001**
Posters/printed information outside of school	49 (23)	79 (34)	+11%	**0.017**
Social media	50 (24)	66 (28)	+4%	0.306
Medical/public health sources	2 (1)	9 (4)	+3%	0.051
I do not see or hear information about cleaning hands	5 (2)	4 (2)	No change	0.741
Attitudes				
Like to wash hands with soap and water	201 (96)	231 (99)	+3%	0.086
Like to clean hands with hand sanitizer	189 (90)	183 (78)	−12%	**<0.001**
Easy to clean hands at school with soap and water	190 (91)	222 (95)	+4%	0.185
Easy to clean hands at school with hand sanitizer	192 (92)	194 (83)	−9%	**0.002**
Practices				
When do you usually clean your hands during the day? (multiple responses)				
Before eating	99 (47)	150 (64)	+17%	**<0.001**
After using the restroom	46 (22)	77 (33)	+11%	**0.011**
After playing	36 (17)	51 (22)	+5%	0.227
After coughing/sneezing	0 (0)	2 (1)	+1%	0.500
After touching pets or animals	8 (4)	9 (4)	No change	0.992
After touching something dirty	33 (16)	61 (26)	+10%	**0.008**
When hands are visibly dirty	5 (2)	2 (1)	−1%	0.263
Do you wash your hands with soap and water at school? (Yes)	207 (99)	232 (100)	+1%	0.605
Do you use hand sanitizer at school? (Yes)	195 (93)	166 (71)	−22%	**<0.001**

COVID-19 = coronavirus disease 2019; KAP = knowledge, attitudes, and practices.

Bold value denotes statistical significance.

* This is not a complete list of questions from the student knowledge, attitudes, and practices (KAP) survey. The selected questions represent the most important KAP concepts that may have been influenced by the intervention.

^†^ *P*-values were calculated using χ^2^ or Fisher’s exact test.

^‡^ Out of 203 responses at baseline and 234 responses at follow up.

^§^ Handwashing with soap and water is the correct hand hygiene method for when hands are visibly dirty.

During both baseline and follow-up, almost all students reported that they like to wash their hands with water and soap (96% and 99%, *P*-value = 0.086). Most students also reported that they like to clean their hands with hand sanitizer, but the proportion was higher at baseline (90%) compared with follow-up (78%) (*P*-value <0.001). Approximately nine of 10 students shared that it is easy for them to clean their hands at schools with soap and water (91% at baseline and 95% at follow-up, *P*-value = 0.185). Similarly, high proportions of students expressed that it is easy to clean hands at school with hand sanitizer at both time points but was higher at baseline (92% at baseline and 83% at follow-up, *P*-value = 0.002).

When students were asked when they usually clean their hands during the day (without providing possible responses), before eating was the most common response at baseline and follow-up (47% and 64%, respectively; *P*-value <0.001), followed by after using the restroom (22% and 33%; *P*-value = 0.011). Almost all students reported that they wash their hands with soap and water at school (99% at baseline and 100% follow-up, *P*-value = 0.605). A higher proportion of students reported using hand sanitizer at school at baseline (93%) compared with follow-up (71%) (*P*-value <0.001).

**Figure 3.**
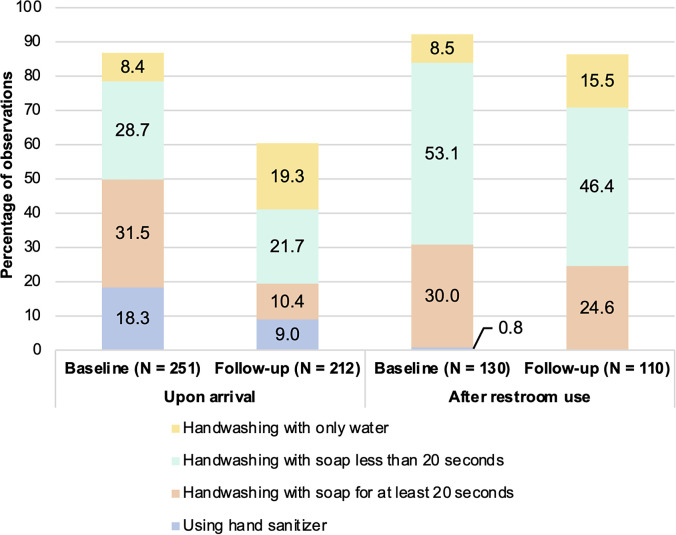
Hand hygiene (HH) methods practiced by students upon arriving to school and after using the restroom at baseline and follow-up assessments. * Handwashing with soap and water for at least 20 seconds is the correct HH method for after using the restroom, whereas hand sanitizer is acceptable upon arrival at school.

A total of 251 and 212 students were observed for HH practices upon their arrival to school during the baseline and follow-up assessment, respectively (Figure 3). At baseline, 87% of students practiced HH upon arriving to school compared with 60% at the follow-up assessment. Hand sanitizer was more commonly used at the entrance at baseline (18%) compared with during follow-up (9%). The percentage of students who washed their hands with soap and water for at least 20 seconds at the school entrance decreased from 32% at baseline to 10% at follow-up.

At school restrooms, 130 students were observed at baseline and another 110 were observed at follow-up. At baseline, 92% of students washed their hands after using the restroom compared with 87% at follow-up. The proportion of students washing hands with soap and water for at least 20 seconds after using the restroom decreased from 30% at baseline to 25% at follow-up.

Overall, 76% of students <10 years old washed their hands with soap after using the restroom compared with 80% of students ≥10 years old (Table 4). Almost equal proportions of male students (76%) and female students (80%) washed their hands with soap after using the restroom. When an HH attendant was present, handwashing with soap after using the restroom was significantly more likely compared with when an attendant was not present (OR = 3.46, 95% CI = 1.11–10.73). Compared with baseline, the odds of handwashing with soap after using the restroom were significantly lower at follow-up (OR = 0.42, 95% CI = 0.21–0.84). When adjusting for the presence of an HH attendant, the odds of handwashing with soap after using the restroom were still significantly lower at follow-up compared with baseline (adjusted OR [aOR] = 0.47, 95% CI = 0.24–0.93).

**Table 4:** Characteristics of students/hygiene stations and their association with handwashing (HW) with soap and water* after using the restroom at baseline and follow-up

	Bivariate			Multivariable		
	Total Students	HW with Soap and Water *n* (%)	OR (95% CI)	*P*-Value	aOR (95% CI)	*P*-Value
Age						
<10 years old	131	99 (76)	Ref	–	–	–
>10 years old	108	86 (80)	1.42 (0.73–2.76)	0.297	–	–
Sex						
Male	132	100 (76)	Ref	–	–	–
Female	106	85 (80)	1.30 (0.68–2.49)	0.435	–	–
Locality						
Urban	60	46 (77)	Ref	–	–	–
Rural	180	140 (78)	1.25 (0.26–6.01)	0.778	–	–
Presence of hand hygiene attendant						
No	184	135 (73)	Ref	–	Ref	–
Yes	56	51 (91)	3.46 (1.11–10.73)	**0.032**	3.05 (0.92–10.13)	0.068
Region						
North	62	43 (69)	Ref	–	–	–
Central	114	88 (77)	1.81 (0.50–6.52)	0.367	–	–
South	64	55 (86)	2.92 (0.68–12.45)	0.148	–	–
Assessment time point						
Baseline	130	108 (83)	Ref	–	Ref	–
Follow-up	110	78 (71)	0.42 (0.21–0.84)	**0.014**	0.47 (0.24–0.93)	**0.030**

aOR = adjusted odds ratio; HW = handwashing; OR = odds ratio; Ref = referent group.

Bold value denotes statistical significance.

* This analysis investigates handwashing with soap after using the restroom; however, handwashing with soap for at least 20 seconds is the recommended hand hygiene method.

**Figure 4.**
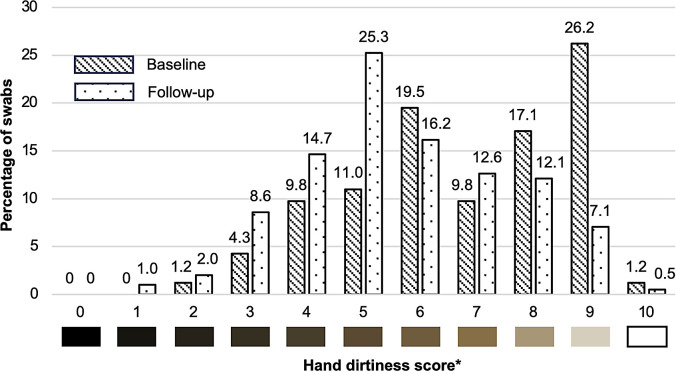
Distribution of hand dirtiness score at baseline (*n* = 164) and follow-up (*n* = 198). * The color density under each score corresponds to the color density of the Quantitative Personal Hygiene Assessment Tool (qPHAT) scale.

A total of 362 hand dirtiness swabs were collected among students, 164 at baseline and 198 at follow-up (Figure 4). The overall median from all hand dirtiness swabs was six (range: one to 10). The median hand dirtiness scores at baseline and follow-up were seven (range: two to 10) and five (range: one to 10), respectively. A lower score corresponds to more visible debris on the swab. Among all hand dirtiness assessment participants, 58% of students from standards I–III had a swab with a score ≥6, whereas 62% of students from standards IV–VI had a score ≥6 (Table 5). A higher proportion of female students (68%) who participated had a score ≥6, compared with 51% of male students. In both the bivariate and multivariable analyses, sex, geographical region of the school location, and assessment time point were significant predictors of HH dirtiness scores. Female students had higher odds of a score ≥6 compared with male students (aOR = 2.55, *P*-value <0.001). Students in the South (aOR = 0.11, *P*-value = 0.007) and students participating in the follow-up assessment (aOR = 0.31, *P*-value <0.001) had lower odds of having a score ≥6 compared with students in the North and students in the baseline assessment, respectively. There was no association between KAP scores and hand dirtiness level.

**Table 5:** Associations of student hand dirtiness assessment scores with student/school characteristics and KAP scores

	Bivariate			Multivariable		
	Total Students	qPHAT >6 (%)*	OR (95% CI)	*P*-Value	aOR (95% CI)	*P*-Value
Grade						
Standards I–III	175	101 (58)	Ref	Ref	–	–
Standards IV–VI	187	116 (62)	1.13 (0.70–1.84)	0.616	–	–
Sex						
Male	173	88 (51)	Ref	Ref	Ref	Ref
Female	189	129 (68)	2.78 (1.67–4.63)	**<0.001**	2.55 (1.51–4.30)	**<0.001**
Region						
North	122	97 (80)	Ref	Ref	Ref	Ref
Central	117	69 (59)	0.46 (0.09–2.35)	0.352	0.33 (0.07–1.67)	0.181
South	123	51 (41)	0.13 (0.03–0.66)	**0.014**	0.11 (0.02–0.56)	**0.007**
Locality						
Urban	63	31 (49)	Ref	Ref	–	–
Rural	299	186 (62)	2.18 (0.25–19.06)	0.481	–	–
Knowledge composite score^†^						
Below median	38	26 (68)	Ref	Ref	–	–
At least median	324	191 (59)	0.87 (0.38–1.98)	0.744	–	–
Attitude composite score^†^						
Below median	102	58 (57)	Ref	Ref	–	–
At least median	260	159 (61)	1.17 (0.68–2.03)	0.564	–	–
Practices composite score^†^						
Below median	137	80 (58)	Ref	Ref	–	–
At least median	225	137 (61)	1.31 (0.79–2.17)	0.294	–	–
Have you cleaned your hands today?^†^						
No	20	12 (60)	Ref	Ref	–	–
Yes	342	205 (60)	1.01 (0.37–2.80)	0.980	–	–
Assessment time point						
Baseline	164	121 (74)	Ref	Ref	Ref	Ref
Follow-up	198	96 (48)	0.29 (0.17–0.50)	**<0.001**	0.31 (0.18–0.54)	**<0.001**

aOR = adjusted odds ratio; KAP = knowledge, attitudes, and practices; OR = odds ratio; qPHAT = Quantitative Personal Hygiene Assessment Tool; Ref = referent group.

Bold value denotes statistical significance.

* On the Quantitative Personal Hygiene Assessment Tool scale, a higher score corresponds to less visible debris on the swab.

^†^ Student knowledge, attitudes, and practices score/response for students with a Quantitative Personal Hygiene Assessment Tool swab.

## DISCUSSION

Our study aimed to evaluate the HH behavior change interventions through assessments of HH knowledge, attitudes, practices, and direct observations of HH practices and hand dirtiness among primary school students in 12 pilot schools in Belize before and after a behavior change intervention. Although the overall knowledge or attitudes did not change after the intervention, the study identified some positive results: a higher proportion of students at follow-up compared with baseline identified the correct reasons for handwashing and self-reported practicing HH during some critical moments, such as before eating and after using the restroom. Contrary to the expectation, observed handwashing with soap after restroom use decreased significantly from baseline to follow-up. The median hand dirtiness score decreased from baseline to follow-up, indicating a higher proportion of swabs with higher density of visible dirt at follow-up.

Because of the short time frame between the lesson implementation and the follow-up assessments, the HH perceptions and practices observed at follow-up may be inflated and may wane over time. It is difficult to determine if the intervention will result in long-term changes in HH awareness and practices. To cultivate behavior changes, consistent and repeated messaging may be beneficial, although messages need to be repeated through creative methods to retain interest. Other WASH-in-school interventions have integrated multiple educational and communication techniques to promote knowledge and behavior change.^7^^,^^16^ Although the timing was a challenge, the workshop allowed administrators from different areas of the country to collaboratively design interactive lessons specifically for HH. For some grade levels, there were no specific learning outcomes requiring the teaching of hygiene; thus, this exercise encouraged the administrators to creatively integrate hygiene content into different subject areas for those grade levels.

Although the intervention did not positively change HH behaviors during the study period, external factors may have negatively impacted the outcomes. One major change between the baseline and the follow-up assessments was the scale-back of COVID-19–mandated measures at schools. The baseline assessment was conducted after Belize had recovered from its largest wave of COVID-19,^17^ whereas the follow-up evaluation was conducted after the WHO had declared that COVID-19 was no longer an international public health emergency.^18^ At baseline, schools had recently reopened and students in Belize were required to wash their hands or use hand sanitizer before entering the school compound; however, this protocol was no longer mandated during the follow-up assessment. At the baseline assessment, students spent the entire school day in the classroom, whereas at follow-up, students were allowed to play in the schoolyard before school and during breaks. When asked about the importance of handwashing, COVID-19 decreased as a reported reason, from 18% at baseline to 3% at follow-up. The reduced focus on COVID-19 precautionary measures at the national and local levels, along with the change in students’ awareness and perceived risk of infection, potentially played a role in students’ HH perceptions and practices at school. A previously published review including studies from 30 countries around the world found an association between risk perception and abiding by public health measures during the pandemic.^19^ The changes in school protocols and individual perceptions likely contributed to the decrease in HH practices and increase in hand dirtiness at follow-up. Although our facility assessment at the pilot schools showed a decrease in HH resources between baseline and follow-up,^11^ this was unlikely to be a contributing factor to the decrease in HH practices as direct observations of student HH practices were conducted in areas with access to HH stations.

There have been inconsistent results on the impact of WASH interventions in schools. Similar implementation of environmental nudges in schools in Bangladesh and the Philippines resulted in an increase in handwashing among students.^20^^,^^21^ In contrast, a randomized trial in Laos showed no effects of improved WASH services and education on student absences and reported health outcomes.^22^ Another trial in Chinese primary schools also found no impact of WASH in school programs on reported respiratory and gastrointestinal symptoms by students.^23^ Because intervention setting, fidelity, and user acceptability play a significant role in the outcomes, it is not unexpected that our intervention in Belize did not yield a positive change in HH behaviors during a waning pandemic.

To our knowledge, this was the first study to systematically assess student HH knowledge, attitudes, and practices in schools in Belize, especially during a public health emergency where improved HH was recommended. Our comprehensive assessments used multiple data collection instruments, which allowed us to understand HH knowledge, attitudes, and practices from different perspectives. The comprehensive assessments in our study allowed us to triangulate student knowledge, attitudes, and self-reported hygiene practices with actual HH practices. Furthermore, our study included schools with very different geographical and social contexts across the six districts of Belize. The schools selected also varied in size and management systems, as some were operated entirely by the government, whereas others belonged to a religious denomination and received support from the government. However, because the pilot schools were selected on self-reported gaps in HH resources, selection bias may have been introduced. Nevertheless, the sampling techniques used in the in-person assessments enhanced the representation of students across ages and sex at the pilot schools.

There are other limitations worth noting about the intervention and the data collection process. Unfortunately, we did not have data on access to HH resources at other schools in the country at the time of the baseline evaluation; therefore, we could not make comparisons. Also, the implementation of the interactive HH lessons varied by school. Because the lesson plan development workshop was hosted very close to the end of the school year, the lessons were not implemented for every grade level at every pilot school because of other end-of-year activities. The installation of the nudges was completed at the schools at different times during the intervention period because of difficulty in selecting the appropriate materials. Though the students encountered the nudges daily, they may not be aware of the purpose of each of the nudges. For example, they may have viewed the pavers with the painted footsteps as marks for where they should stand when lining up to wash hands as opposed to seeing them completing the path from the toilet to the handwashing station. Furthermore, because there were existing environmental nudges related to COVID-19 prevention measures at some schools, students may not have distinguished that our nudges were targeting HH. Changes to the design of the HH nudges may be necessary to improve their effectiveness. Providing HH messages in Spanish and/or other dialects of the local community may increase impact among students whose primary language is not English. Additionally, the painted arrow pointing to the soap was meant for soap to be placed by the handwashing station; however, some schools preferred to keep the soap bottles in the classroom to prevent theft or students playing with and wasting the soap. Future studies may consider strengthening the monitoring and evaluation period to collect data on program fidelity and implement adaptive changes.

Although the KAP survey was extensively reviewed to ensure the language was appropriate for primary school students, students from the lower grades may not have completely understood the questions. The survey was offered both in English and Spanish, but some students were more comfortable in other native languages (e.g., Mayan Q’eqchi’), which could have influenced their understanding of the questions and their responses. There may also be response and desirability biases in the attitudes and self-reported practices section of the KAP survey if the students responded with what they believed were the correct responses instead of sharing their true HH perceptions, attitudes, and practices. Because the KAP survey was developed for this study, there is no previous data on the internal reliability of the questions. Furthermore, the assumption of sex and age of the students during the HH observations was subjective to the enumerators’ observation so misclassification could have occurred. The assessments at baseline and follow-up only included 11 schools as one school was not included at baseline, and another was not included at follow-up. Although the analysis assumed independent observations, the baseline and follow-up participants may not have been independent as some students may have participated in both time points. Furthermore, only two of the 12 schools were classified as urban; thus, the results of this study may not be generalizable to all urban schools in Belize. Lastly, the pre–post intervention design of the study without the presence of a control group may have muddled the impact of the intervention and pandemic-related changes.

Long-term interventions and monitoring are critical to foster behavior changes. School-based interventions should be tailored based on the environmental, social, and cultural context of the schools and the community. Behavior change is a complex phenomenon requiring a multifaceted intervention. A systematic review shows that health interventions among children ages 5 to 11 commonly include techniques such as involvement of the caregiver and use of behavioral and interactive tools.^24^ Future interventions should consider a combination of behavioral drivers from multiple levels, including psychological, societal, and environmental.^25^ In addition to gathering suggestions at the baseline assessment, feedback from school staff and students can be extremely valuable in selecting and adapting the appropriate intervention for their school settings. Additionally, HH behaviors can be influenced by a multitude of factors, including beliefs and practices at home and in the community. Community participation has been shown to positively impact the outcomes of WASH interventions in low- and middle-income countries.^26^ Future school-based HH initiatives can involve families and community members. In addition to the interventions at schools, promoting HH practices at different levels in the community, such as the household level and community gathering locations, could help improve HH practices and encourage sustained behavior change.

## CONCLUSION

Public health measures related to the COVID-19 pandemic at the national and local levels may have played a substantial role in HH practices at schools. The lack of improvement in student HH adherence after the school-based intervention is most likely because of the scale-back of COVID-19 protocols and reduced perceived risk of infection. Continued HH programs are important to bridge the gaps in HH practices in school settings, especially when pandemic measures are no longer in place. Future studies could consider long-term implementation and evaluation to foster behavior change with school-based interventions.
